# Melamine Impairs Female Fertility via Suppressing Protein Level of Juno in Mouse Eggs

**DOI:** 10.1371/journal.pone.0144248

**Published:** 2015-12-03

**Authors:** Xiaoxin Dai, Mianqun Zhang, Yajuan Lu, Yilong Miao, Changyin Zhou, Shaochen Sun, Bo Xiong

**Affiliations:** College of Animal Science and Technology, Nanjing Agricultural University, Nanjing, 20095, China; Inner Mongolia University, CHINA

## Abstract

Melamine is an organic nitrogenous compound widely used as an industrial chemical, and it has been recently reported by us that melamine has a toxic effect on the female reproductive system in mice, and renders females subfertile; the molecular basis, however, has not been adequately assessed. In the present study, we explore the underlying mechanism regarding how melamine compromises fertility in the mouse. The data showed that melamine exposure significantly impaired the fertilization capability of the egg during *in vitro* fertilization. To further figure out the cause, we analyzed ovastacin localization and protein level, the sperm binding ability of zona pellucida, and ZP2 cleavage status in unfertilized eggs from melamine fed mice, and no obvious differences were found between control and treatment groups. However, the protein level of Juno on the egg plasma membrane in the high-dose feeding group indeed significantly decreased compared to the control group. Thus, these data suggest that melamine compromises female fertility via suppressing Juno protein level on the egg membrane.

## Introduction

Melamine (1,3,5-triazine–2,4,6-triamine, or C3H6N6) is a nitrogen heterocyclic triazine compound [1, 2] which has been widely used as an industrial chemical in many plastics, adhesives, glues, and laminated products such as plywood, cement, cleansers, fireretardant paint, and more [2, 3]. Melamine developed as a chemical in the 1830s, and had varied and widespread legitimate uses. A food safety incident outbroke in China in 2008 which was involved milk and infant formula along with other food materials and components being adulterated with melamine had attracted much attention to the limited usage of melamine [2]. Accumulating evidence has revealed that long-term exposure to melamine could damage the reproductive systems in mammals, and lead to male infertility and fetal toxicity in the rat [4]. Also, previous report by us has shown that melamine feeding renders female mice subfertile [5].

Fertilization is a culminating event in mammals, involved in two haploid cells, the egg and the sperm. They meet in the female reproductive tract, interact, and finally fuse to become a new, genetically distinct, diploid cell [6, 7]. Accomplishment of fertilization needs several sequential steps of gamete interaction: Capacitated sperm bind to the zona pellucida surrounding eggs, and then release acrosomal contents by exocytosis and penetrate the ZP. After that, acrosome-reacted sperm reach, bind to and fuse with the egg membrane to form fertilized egg [8]. ZP is a glycoproteinaceous translucent matrix that surrounds the mammalian eggs and embryos, and plays important roles during oogenesis, sperm-egg binding, fertilization and implantation [9–11]. This matrix of human is composed of four glycoproteins ZP1, ZP2, ZP3, and ZP4, whereas mouse ZP is composed of ZP1, ZP2 and ZP3 (ZP4 being a pseudogene) [9, 12]. In the supramolecular structure model, sperm-binding site is an N-terminal domain of ZP2 that depends on the cleavage status of ZP2 [9, 13]. Subsequent to sperm membrane fusion with oolema, cleavage of ZP2 helps in prevention of polyspermy [14].

Ovastacin is a pioneer component of mammalian cortical granules which belongs to a member of the large astacin family of metalloendoproteases [15, 16]. This oocyte-specific protein is exocytosed from cortical granules triggered by fertilization and is responsible for post-fertilization ZP2 cleavage to block sperm binding and polyspermy. Thus, in ovastacin-deficient mice, albeit fertilization, sperm are still able to bind to zona pellucida surrounding embryos because ZP2 remains intact, which accordingly, renders ovastacin-deficient females subfertile due to the polyspermy [14]. In addition, fetuin-B has been recently identified as the inhibitor of ovastacin, and genetic ablation of fetuin-B causes premature ZP hardening and, consequently, female infertility [17, 18]. This result shows that premature cleavage of ZP2 can result in infertility in mice.

Glycophosphatidylinositol (GPI)-anchored receptors on the egg surface are essential for fertilization because sperm lacking them render eggs infertile [19, 20]. A breakthrough was made in 2014 when Gavin J. Wright’s group identified folate receptor 4 (Folr4) as the Izumo1 receptor, displayed on the surface of egg they named this protein “Juno” after the Roman goddess of fertility and marriage [7]. Juno is expressed on the surface of oocyte, a GPI-anchored protein that is essential for female fertility. Juno-deficient mice are infertile because eggs lacking Juno cannot fuse with normal acrosome reacted sperm [6, 7].

Our most recent report indicates that melamine negatively affects female fertility in mice [5]. Here, we further explore the possible molecular basis at several levels during fertilization, and our data provide the evidence showing that melamine compromises female fertility via regulating the protein level of Juno, rendering eggs non-fusible with sperm.

## Materials and Methods

### Ethic statement

Our study was approved by the Animal Research Institute Committee of Nanjing Agricultural University, China, and all mice were handled in accordance with the Committee guidelines. Mice were housed in a temperature-controlled room with proper darkness-light cycles, fed with a regular diet, and maintained under the care of the Laboratory Animal Unit, Nanjing Agricultural University, China. The mice were euthanized by cervical dislocation.

### Animals and feeding treatment

The female 4-week-old ICR mice were housed in separate cages at controlled condition of temperature (20–23°C) and illumination (12h light-dark cycle), and had free access to food and water throughout the period of the study. After 1 week acclimation to the laboratory environment, mice were randomly assigned to 3 groups (n = 40), with an average body weight of 18 g and were each orally given 0, 10 or 50mg/kg/d of melamine dissolved in water for 8 weeks. The animals were observed each three days, and there were no one ill or dead during administration.

### *In vitro* fertility

Cauda epididymides were lanced in a dish of human tubal fluid (HTF) medium (EMD Millipore, Billerica, MA) to release sperm, followed by being capacitated for 1 hr (37°C, 5% CO2) and added to ovulated eggs at a concentration of 4 x 10^5^/ml sperm in 100μl HTF for 5 hr at 37°C, 5% CO2. The presence of two pronuclei was scored as successful fertilization.

### Immunofluorescent and confocal microscopy

Ovulated eggs were fixed in 4% paraformaldehyde in PBS (pH 7.4) for 30 minutes and permeabilized in 0.5% Triton-X-100 for 20 min at room temperature. Then, oocytes were blocked with 1% BSA-supplemented PBS for 1 h and incubated at 4°C overnight or at room temperature for 4 h with rat monoclonal anti-mouse folr4 antibody (1:100, BioLegend, CA) or rabbit polyclonal anti-mouse ovastacin antibody (1:100, obtained from Dr. Jurrien Dean). After washing four times (5 min each) in PBS containing 1% Tween 20 and 0.01% Triton-X 100, eggs were incubated with an appropriate secondary antibody for 1 h at room temperature. Alexa Fluor 555 donkey anti-rabbit IgG (H+L) was obtained from Invitrogen (Carlsbad, CA). After washing three times, eggs were stained with PI or Hoechst 33342 (10 μg/ml) for 10 min. Finally, eggs were mounted on glass slides and viewed under a confocal laser scanning microscope (Carl Zeiss 700).

### Western blot analysis

Ovulated eggs or two-cell embryos were lysed in 4× LDS sample buffer with 10× reducing reagent (Life Technologies-Invitrogen) and heated at 100°C for 5 min. Proteins were separated on 12% Bis-Tris precast gels, transferred to PVDF membranes, blocked in 5% nonfat milk in TBS (Tris buffered saline, pH 7.4) with 0.1% Tween 20 (TBST) for 1 hr at room temperature, and then probed with 1:500 dilution of M2c.2 antibody (obtained from Dr. Jurrien Dean) at 4°C overnight. After washing three times in TBST (10 min each), blots were incubated 1 hr with a 1:10,000 dilution of HRP (Horse Radish Peroxidase) conjugated goat anti-rabbit IgG (Santa cruz, Texas). Chemiluminescence was performed with ECL Plus (Piercenet) and signals were acquired by Tanon-3900.

### Sperm binding assay

Caudal epididymal sperm were isolated from wild-type ICR mice and placed under oil (Sigma-Aldrich, MO) in HTF medium previously equilibrated with 5% CO2 and capacitated by an additional 1 hr of incubation at 37°C. Sperm binding to ovulated eggs or two-cell embryos isolated from control and melamine-treated mice was observed using capacitated sperm and control two-cell embryos as a negative wash control. Samples were fixed in 4% PFA for 30 min, stained with Hoechst 33342. Bound sperm were quantified from z projections acquired by confocal microscopy, and results reflect the mean ± S.E.M. from at least three independently obtained samples, each containing 10–12 mouse eggs/embryos.

### Statistical analysis

The data were expressed as mean ± SEM and analyzed by one-way ANOVA, followed by LSD’s post hoc test, which was provided by SPSS16.0 statistical software. The level of significance was accepted as p<0.05.

## Results

### Melamine exposure compromises the *in vitro* fertilization

**Fig 1:**
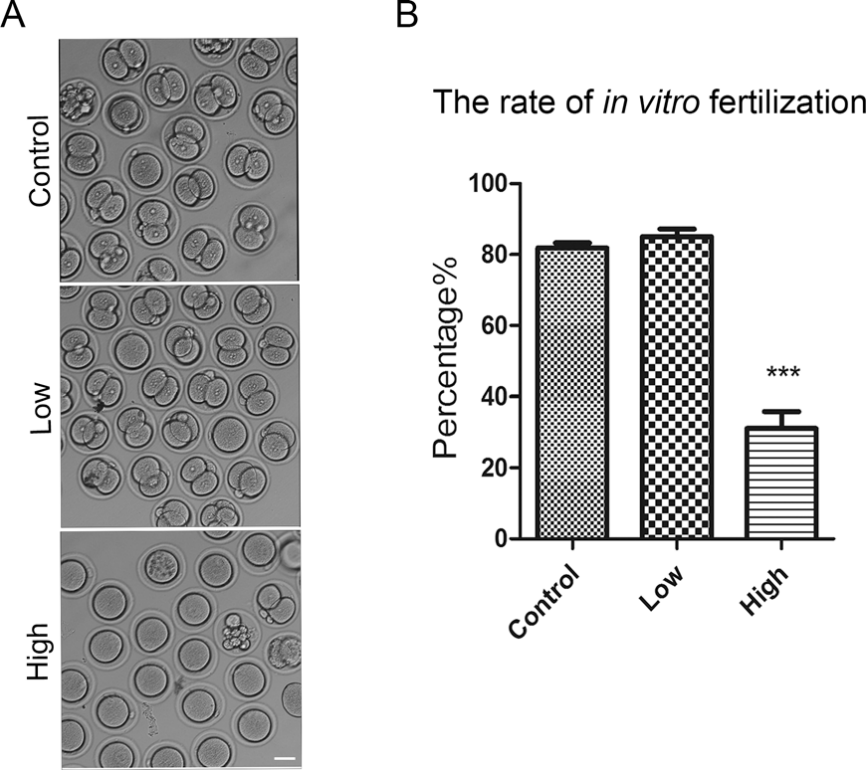
*In vitro* fertilization of ovulated eggs from melamine fed mice. (A) Representative images of fertilized eggs in control and melamine-treated mice. Most of eggs were not fertilized in high-dose treatment group. Scale bar, 40μm. (B) *In vitro* fertilization rates, control oocytes (n = 91), low group (n = 112), and high group (n = 124). Fertilization was determined by the presence of 2 pronuclei 12 hr after insemination. Data were presented as mean percentage (mean ± SEM) of at least three independent experiments. Asterisk denotes statistical difference at a P < 0.05 level of significance.

Melamine feeding model was set up by eight-week diet, and was classified into control (0mg/kg/d), low-dose (10mg/kg/d) and high-dose (50mg/kg/d) groups. To confirm the *in vivo* fertility result reported previously, eggs from three groups were collected and used for *in vitro* fertilization, respectively. As shown in Fig 1, the fertilization rate of low-dose group is comparable to that of control group (85.0 ± 2.2% VS 81.9 ± 1.4%), but the rate of how-dose group is significantly lower than and low dose (50mg/kg/d) group, the high dose (50mg/kg/d) group was significantly decreased (P < 0.01). Because there was no obvious defect on the fertilization in low-dose group, we only compared control and high-dose groups in below experiments.

### Melamine does not result in mislocalization and premature exocytosis of ovastacin in eggs

**Fig 2:**
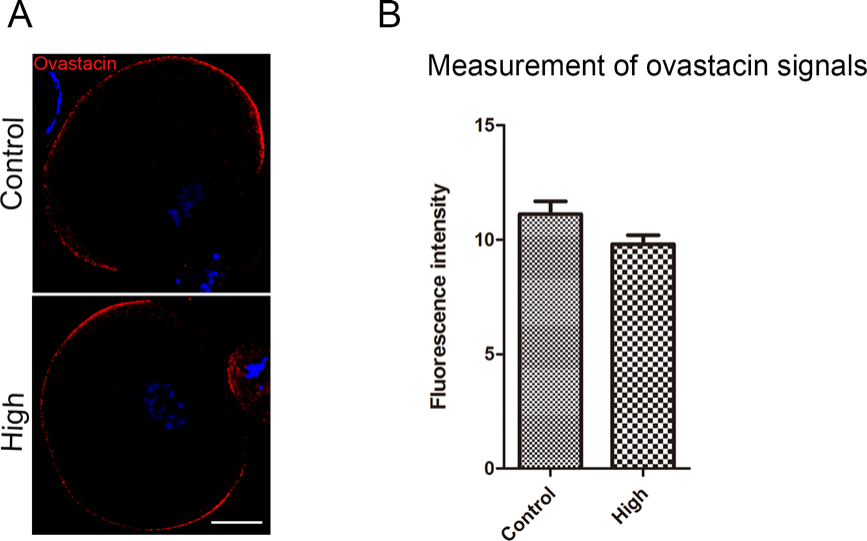
Effect of melamine feeding on the ovastacin localization and protein level in ovulated eggs. (A) Ovastacin was stained with rabbit polyclonal anti-mouse ovastacin antibody and examined by confocal microscopy. Scale bar, 20μm. (B) Measurement of fluorescent intensity of ovastacin signals. There was no significant difference of ovastacin signals between control (n = 15) and treatment (n = 15) groups. Data were presented as mean percentage (mean ± SEM) of at least three independent experiments. Asterisk denotes statistical difference at a P < 0.05 level of significance.

To determine the possible reason causing the failure of fertilization, we first examined the localization and protein level of ovastacin, an oocyte-specific metalloprotease in the cortical granules which is responsible for post-fertilization cleavage of N-terminus of ZP2, the sperm binding site in the zona pellucida, to block polyspermy, because mislocalization and premature release of ovastacin before fertilization in unfertilized eggs would lead to zona hardening so that compromise the fertilization. To validate this, we performed the immunostaining of ovastacin under the same condition in control and melamine fed groups, and measured the immunofluorescent intensity. As shown in Fig 2, ovastacin was localized under the oocyte subcortical region and excluded in cortical granule free domain, and both localization and signal intensity of ovastacin were comparable between control and high-dose groups (11.1 ± 0.6% VS 9.9 ± 0.4%), indicating that melamine would not lead to the mislocalization and premature exocytosis of ovastacin, which might be one of the factors leading to the fertilization failure.

### Melamine does not affect sperm binding ability on eggs

**Fig 3:**
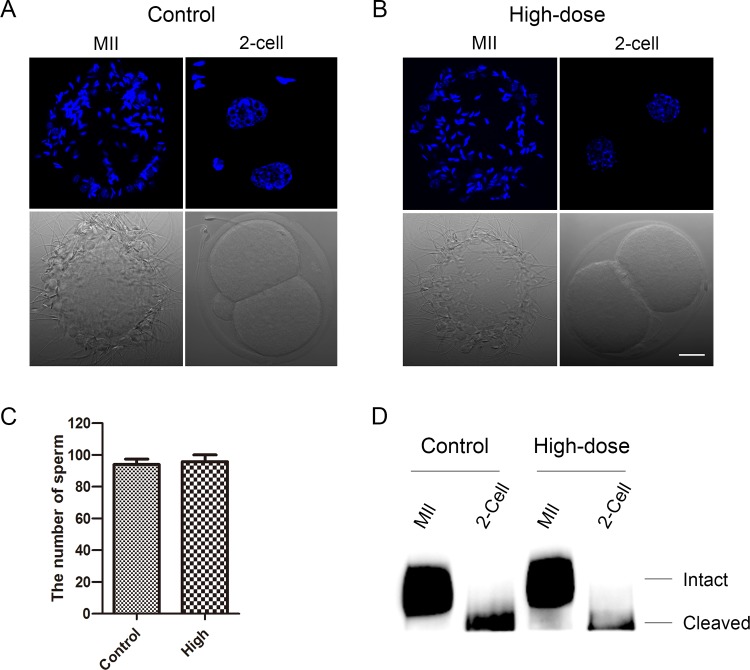
Sperm binding capability and ZP2 cleavage status. (A, B) Eggs and two-cell embryos from control and melamine fed mice were incubated with capacitated sperm for 1 hr. After washing with a wide-bore pipette to remove all but two to six sperm on normal two-cell embryos (negative control), eggs and embryos with sperm were fixed and stained with Hoechst 33342. Scale bar, 20μm. (C) The number of sperm binding to the surface of zona pellucida surrounding eggs. There was no significant difference between control (n = 10) and high-dose (n = 8) groups. (D) Western blot analysis of ZP2 cleavage status in eggs and two-cell embryos using M2c.2 antibody that recognizes the C-terminal domain of ZP2. The size of intact ZP2 is 120 kD, and the size of cleaved C-terminal fragment of ZP2 is 90 kD.

Next, we tested if melamine has effect on the sperm–zona pellucida binding *in vitro*. Based on the fact that sperm bind to the N-terminus of ZP2 in unfertilized eggs but not 2-cell embryos in which has been cleaved by ovastacin released by cortical granules, we set up 2-cell embryos as the negative control for the sperm binding assay. The immunofluorescent analysis showed that the number of sperm binding to the surface of zona pellucida surrounding eggs from both control and high-dose groups is comparable (94.0 ± 3.5% VS 95.8 ± 4.3%) (Fig 3A, 3B and 3C), suggesting that impairment of the fertilization capability of eggs by melamine does not result from the zona binding defect. Because sperm binding to zona is determined by the cleavage status of ZP2, we also performed the western blot using the antibody M2c.2 which recognizes the C-terminus of mouse ZP2. In the control group, ZP2 remained intact in unfertilized eggs and cleaved in 2-cell embryos as expected (Fig 3D). In high-dose group, there was no ZP2 cleavage detected in unfertilized eggs, consistent with the above result that sperm binding ability is normal in melamine-treated eggs.

### Melamine exposure reduces Juno protein level on the egg membrane

**Fig 4:**
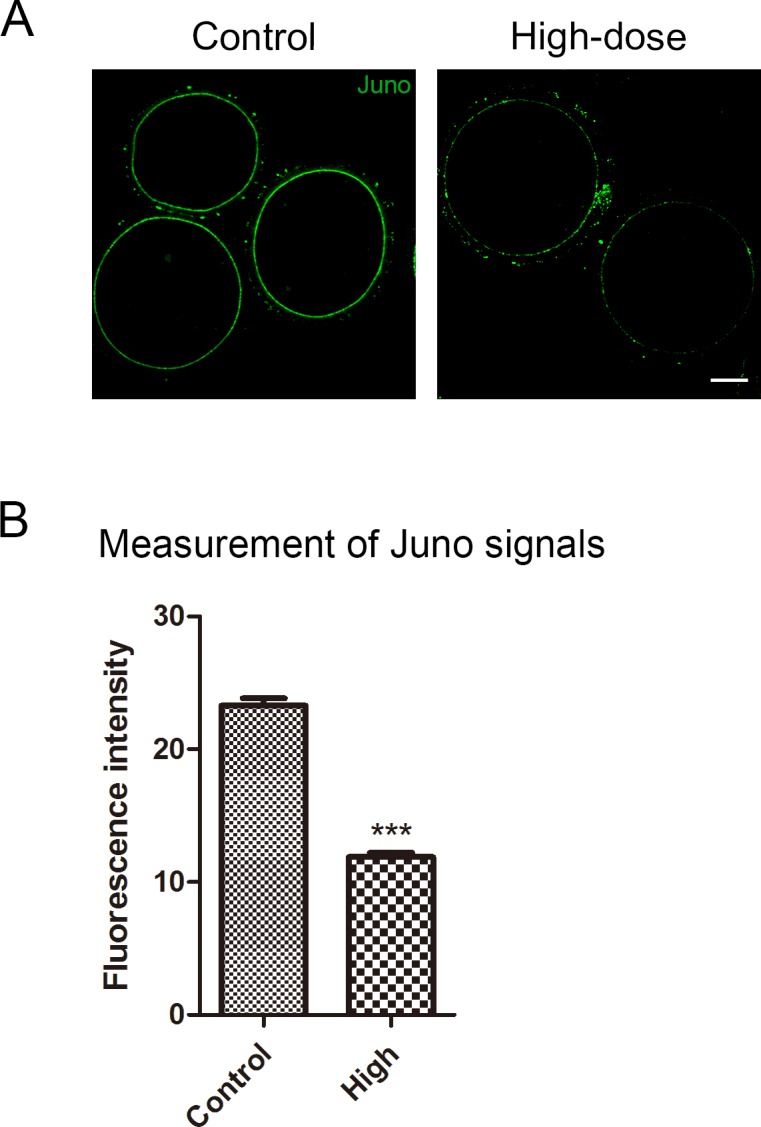
Effect of melamine feeding on the protein level of Juno in ovulated eggs. (A) Juno was stained with rat monoclonal anti-mouse folr4 antibody and examined by confocal microscopy. Scale bar, 20μm. (B) Measurement of fluorescent intensity of Juno signals. The protein level of Juno in egg membrane was significantly reduced in high-dose group (n = 15) compared to control group (n = 25). Data were presented as mean percentage (mean ± SEM) of at least three independent experiments. Asterisk denotes statistical difference at a P < 0.05 level of significance.

Since we have already ruled out the defect on the zona pellucida, we further explored the possible candidates on the egg membrane. Juno is a recently-found receptor on the egg membrane which binds to Izumo1 in the sperm head to mediate the sperm-egg fusion. We performed the immunostaining of Juno, and found that it was evenly distributed on the egg membranes (Fig 4A). Furthermore, we measured the immunofluorescent intensity of egg membranes in control and high-dose groups, and the result showed that the protein level of Juno in the high-dose group was remarkably lower than that in control group (11.6 ± 0.3 VS 24.2 ± 0.6, P < 0.01, Fig 4B). Thus, the subfertility phenotype induced by melamine is probably caused by the decreased level of Juno on the egg membrane.

## Discussion

Melamine (2, 4, 6-triamino-1, 3, 5-triazine), a chemical material, is a widely used industrial chemical that is not considered acutely toxic with a high LD_50_ in animals, and the oral LD_50_ ranges from 3.2 g/kg to 7.0 g/kg in mice [21]. However, long-term exposure to melamine could lead to infertility in rat males [4]. Also, our most recent report indicates that melamine negatively affects female fertility in mice [5].

In the present study, we further investigated the possible molecular mechanism regarding the effect of melamine on the fertility of female mice. We found that feeding mice with the melamine-contained diet had no effects on the ovastacin localization and exocytosis, as well as the sperm-zona pellucida binding, but indeed compromised the Juno protein level on the egg membrane, which might be the major cause leading to the female subfertility.

Fertilization is a unique and multi-step event that initiates the onset of development. During mammalian fertilization, capacitated sperm must bind to and penetrate the specialized extracellular matrix of the egg, known as zona pellucida, and then fuse with the oolemma to become the fertilized eggs [8]. The mouse genetic studies have defined the N-terminal domain of ZP2 as the sperm binding site in the zona pellucida, and the sperm binding is determined by the ZP2 cleavage status independent of fertilization [9]. Following fertilization, ZP2 undergoes proteolytic cleavage by an oocyte-specific astacin-like metalloendoprotease, first reported as ovastacin (citation), released from the cortical granules, and sperm no longer bind to mouse embryos [22]. However, if ovastacin is exocytosed during oogenesis or oocyte maturation before fertilization, it will prematurely cleave the N-terminus of ZP2 and result in less or no sperm binding, leading to the fertilization failure. Based on these understandings, we examined the possible reasons that would cause the melamine-induced female subfertility in mice one after another. Normal localization and protein level of ovastacin in melamine exposed eggs indicated that impairment of fertility is not due to the defect of ovastacin. Next, we tested the sperm binding to zona pellucida. Both sperm binding assay and western blot analysis of ZP2 cleavage revealed that melamine does not result in the zona defect.

Juno is an essential cell-surface protein as the receptor for Izumo1 on the plasma membrane of mouse eggs [7]. Juno and Izumo1 play crucial role in sperm-egg fusion in mice [7, 23, 24]. In other words, both Juno-deficient females and Izumo1-deficient males are infertile because their gametes cannot fuse to their wild-type partner’s cells. Therefore, we aimed Juno as our next candidate. Our immunostaining and signal measurement results showed that high-dose (50mg/kg/d) feeding of melamine to female mice led to significant decrease of Juno protein level on the plasma membrane of unfertilized eggs. This finding consistently interprets the subfertility phenotype, because the small amount of remaining Juno renders some melamine-exposed eggs still fertilizable.

Taken together, we present data here to demonstrate that melamine negatively affects female fertility through suppressing Juno protein level in mice. As for how melamine regulates its protein expression or degradation, it needs the more in-depth investigation in the future.
